# Editorial: Microbiological food safety: Assessment of processing, storage, and transportation

**DOI:** 10.3389/fmicb.2023.1184341

**Published:** 2023-03-28

**Authors:** Anil Kumar Puniya, Utkarsh Jain, Harmeet Singh Dhillon, Jen-Yi Huang, Navin Rastogi, Buddhiman Tamang

**Affiliations:** ^1^Dairy Microbiology Division, ICAR-National Dairy Research Institute, Karnal, India; ^2^Amity Institute of Nanotechnology, Amity University Uttar Pradesh, Noida, India; ^3^Department of Food Science, Purdue University, West Lafayette, IN, United States; ^4^Department of Food Engineering Central Food Technological Research Institute, Mysore, Karnataka, India; ^5^Department of Microbiology, Sikkim University, Sikkim, India

**Keywords:** microbiology, food, safety, processing, storage

Identifying tomorrow's “Microbiological Food Safety” front-runners is critical for protecting future innovation and research in microbiology. This editorial showcases outstanding research conducted by academic scholars in their respective fields. Our objective is to emphasize the contributions of the next generation of leaders in the realm of “Microbiological Food Safety”, and their innovative theoretical, experimental, and methodological approaches to tackling challenging research issues.

Wages et al. demonstrated the efficacy of neutralizing buffered peptone water (nBPW) in whole-bird carcass-rinsate collections for the recovery of *Salmonella spp*. This study aimed to investigate the effect of BPW and nBPW on the microbes and downstream-culturing techniques. The researchers rinsed carcasses in either BPW or nBPW and analyzed the resulting rinsates for the prevalence of *Salmonella, Enterobacteriaceae*, and *Campylobacter spp*. as well as total *Enterobacteriaceae* and aerobic bacterial load. The study revealed that the type of rinstae influenced aerobic bacterial load and prevalence of *Campylobacter spp*. did not have any effect on the *Salmonella spp*. and *Enterobacteriaceae* prevalence and quantity. Furthermore, a variance in microbiome diversity was found in the downstream analyses of the two rinsate types. The findings suggested that the choice of rinsate buffer used in the collection of whole-bird carcass-rinsate can lead to proportional shifts in the microbiome, resulting in differences in cultured-microbial populations.

Shi et al. conducted a study that investigated the survival of *Salmonella* in dried teas of four different types, which were stored at varying temperatures and subjected to different brewing conditions. The study revealed that *Salmonella* could survive in dried-teas for more than three-months, at temperature ranges varying from 4–25°C. *Salmonella* survival was highest in the teas which were kept at 4°C during storage and were lowest in teas kept at 25°C. Although brewing at the temperature of 75°C and 100°C decreased the number of viable *Salmonella* significantly, the study also discovered that viable but not culturable *Salmonella* developed when brewed at 75°C. The study implies that traditional beliefs about the low risk of dried teas causing salmonellosis may not hold true and emphasized on the potential danger of *Salmonella* contamination in dried teas.

The mechanism behind the tolerance of *Vibrio parahaemolyticus* to high-pressure processing was investigated in a paper by Feng et al.. The researchers used seafood products to isolate 183 *V. parahaemolyticus* strains and identified strain-C4, which carried the gene for virulence factor namely thermostable direct hemolysin. Using adaptive laboratory evolution, they obtained strain N11, which could withstand high-pressure processing of nearly 200 MPa for 10 min. They also observed that the N11 strain exhibited increased Na+/K+-ATPase and catalase activity while upholding the intact structure of cell membrane under high-pressure processing. Additionally, both N11 and C4 strains were pathogenic in mice, causing physiological impacts and damaging spleen cells and the liver. Mutated nucleotides (19) were found during the comparative genomics of the N11 strain, causing a sustained high expression of mlaD and mlaC genes involved in pathogenicity and high-pressure stress-response of *V. parahaemolyticus*. The conclusions of this study highlighted the potential health risks associated with pressure-tolerant foodborne pathogens.

Wu et al. compared the microbial composition, mastitis pathogens, and antibiotic resistance genes (ARGs) in raw milk from farms using different bedding materials—rice husk, sand, and recycled manure solids. Their findings revealed that the bedding material influenced the microbial composition of milk. Milk from the cows housed on sand bedding did not show any mastitis pathogens. The ARGs were in the largest proportion in the sand bedding group and recycled manure solids bedding had the lowest ARGs. However, the use of recycled manure solids bedding might possess a risk to dairy cows' breast health, despite having the lowest content of ARGs in milk.

In their paper on the management and control of bacterial species in fermented foods, Hamamoto et al. developed a PCR-based methodology for identifying the specific strain of *Enterococcus faecalis* i.e., EF-2001. By comparing the sequence of this strain to publicly available whole genome sequences of other strains of *E. faecalis*, they were able to identify unique sequences specific to EF-2001. Using these sequences, they designed primer sets for PCR amplification. The PCR examination demonstrated that the DNA fragments were solely intensified in the genome of the EF-2001 strain and not in other bacterial strains of the same genus or lactic acid bacteria. Additionally, the primers were competent in amplifying DNA fragments even in bacteria subjected to heat treatment and foods that comprise the EF-2001 strain. This approach presents an uncomplicated and exceptionally precise method to recognize specific fermentation strains, including lactic acid bacteria at the strain level, which is crucial for regulating the quality of fermented foods. Their approach is an indispensable resource for overseeing and directing bacterial species in the production of fermented foods.

Zhang et al. investigated the effect of malondialdehyde (MDA), a reactive carbonyl compound that arises from the oxidation of food lipids, on *Lactiplantibacillus plantarum* and *Staphylococcus xylosus* isolated from the fishes that were dried and cured. The researchers observed that the MDA inhibited the growth of *L. plantarum* and *S. xylosus* at the respective minimum inhibitory concentrations of 90 μg/ml and 180 μg/ml. Concentration-dependent antibacterial effects of MDA were observed in time-kill curves. The study also found that MDA caused depolarization of the cell membrane, damage to the cell wall, leakage of Mg2+ and Ca2+, and decreased intracellular ATP leading to intracellular biomolecule modifications and morphological cellular damage. These results demonstrate that MDA has potent antimicrobial properties and suggest the need for measures to prevent and control the oxidation of lipids and microbiological food contamination. The study also contributes to our understanding of the impact of lipid oxidation on microorganisms.

Li et al. conducted a study to develop a fast and efficient method for distinguishing various species of boletes, known for their nutrition, taste, and distinct flavor. They analyzed eight species of boletes comprising 1,707 samples using original MIR spectroscopy data for SVM (support vector machine) modeling, as well as 11,949 spectral images for Alexnet and Resnet modeling. Overall, they produced 15 models for identifying bolete-species. The findings revealed that SVM modeling was not ideal for processing large sample sizes due to its time-consuming nature and lower accuracy. On the other hand, synchronous 3-DCOS and 2-DCOS (dimensional correlation spectroscopy) datasets demonstrated the finest results in the modeling, whereas 1D MIR spectrum dataset had the poorest results. Notably, Resnet modeling on the 2DCOS synchronous dataset yielded the most effective modeling outcomes. The authors concluded that combining deep learning with 3DCOS and 2DCOS spectral imaging can precisely recognize the species of bolete and can potentially aid in identifying other food and Chinese herbal medicine species.

In recent years, there has been a global emphasis on ensuring the safety and nutritional quality of food products, as well as the use of anti-microbial preservatives. Consequently, this “Research Topic” included original research and reviews that explored the production, processing, safety, and storage of food from agricultural and aquaculture systems. Our goal is to inspire future researchers to pursue these important areas of study, with the hope that their work will lead to promising and innovative directions for the future. The publication of “*Microbiological Food Safety: Assessment of Processing, Storage, and Transportation*” seeks to contribute to this effort.

## Author contributions

AP, UJ, HD, J-YH, NR, and BT contributed to the writing and editing of the manuscript. All authors contributed to the article and approved the submitted version.

